# Accurately cleavable goat β-lactoglobulin signal peptide efficiently guided translation of a recombinant human plasminogen activator in transgenic rabbit mammary gland

**DOI:** 10.1042/BSR20190596

**Published:** 2019-06-28

**Authors:** Rui Lu, Ting Zhang, Shaozheng Song, Minya Zhou, Lei Jiang, Zhengyi He, Yuguo Yuan, Tingting Yuan, Yaoyao Lu, Kunning Yan, Yong Cheng

**Affiliations:** 1 College of Veterinary Medicine, Yangzhou University, Yangzhou, China; 2 Jiangsu Co-innovation Center for Prevention and Control of Important Animal Infectious Diseases and Zoonoses, Yangzhou, China; 3 School of Nursing, Wuxi Taihu University, Wuxi, China; 4 Institute of Translational Medicine, Medical College, Yangzhou University, Yangzhou, China

**Keywords:** BLG, mammary gland bioreactor, purification, rabbit, rhPA, signal peptide

## Abstract

Poor expression is the key factor hampering the large-scale application of transgenic animal mammary gland bioreactors. A very different approach would be to evaluate the secretion of recombinant proteins into milk in response to a cleavable signal peptide of highly secreted lactoproteins.

We previously reported rabbits harboring mammary gland-specific expression vector containing a fusion cDNA (goat β-lactoglobulin (BLG) signal peptide and recombinant human plasminogen activator (rhPA) coding sequences) expressed rhPA in the milk, but we did not realize the signal peptide contributed to the high rhPA concentration and did not mention it at that time. And the molecular structure and biological characteristics still remain unknown. So, rhPA in the milk was purified and characterized in the present study.

rhPA was purified from the milk, and the purity of the recovered product was 98% with no loss of biological activity. Analysis of the N-terminal sequence, C-terminal sequence, and the molecular mass of purified rhPA revealed that they matched the theoretical design requirements. The active systemic anaphylaxis (ASA) reactions of the purified rhPA were negative. Taken together, these results indicated that the goat BLG signal peptide can efficiently mediate rhPA secretion into milk and was accurately cleaved off from rhPA by endogenous rabbit signal peptidase.

We have reinforced the importance of a rhPA coding region fused to a cleavable heterologous signal peptide from highly secreted goat BLG to improve recombinant protein expression. It is anticipated that these findings will be widely applied to high-yield production of medically important recombinant proteins.

## Introduction

The ability to recombinantly express a protein of interest of sufficient quantity and quality is critical to many fields of biological science, including the production of bio-pharmaceuticals. Bio-pharmaceuticals represent preventative and therapeutic opportunities for a large number of human disorders. To date, over 250 recombinant therapeutic proteins have been approved, and many more are currently in clinical trials [1]. Transgenic animal mammary glands are the perfect hosts for producing therapeutic recombinant human proteins because of their capacity to perform proper folding and assembly of complex proteins and human-compatible glycosylation [2,3]. The key factor hindering the large-scale application of transgenic animal mammary gland bioreactors is the expression level of recombinant proteins, although it is also very important that the recombinant protein can be secreted from mammary epithelial cells into milk while keeping the original recombinant proteins intact. Accordingly, the study and development of innovative methods for the rapid production of recombinant proteins are essential.

In the past few years, a great deal of work concerning optimization of the production of recombinant proteins by transgenic animal mammary glands has concentrated on the promoter [4], enhancer [5], insulator [6,7], codon usage bias [8–10], and selecting for the integration of expression vectors encoding the genes of interest [11–13]. Because the translocation and secretion of recombinant proteins are mediated by signal peptides, a very different approach would be to evaluate the secretion of recombinant proteins into milk via a cleavable signal peptide of a highly secreted lactoprotein.

The signal peptide, which generally contains 15–30 amino acids, directs polypeptides for processing through the secretory machinery of the cell [14,15]. Signal peptides affect the beginning of the protein translation because they are usually located in the N-terminals of the proteins, and the different primary structures of the signal peptides even affect the folding and transport of the proteins [16]. The level of expression of a protein can be modified by replacing a signal peptide, and replacement of native signal peptides with those from proteins that are known to be secreted at high levels can significantly improve secretion levels [17–19]. Although the importance of signal peptides has been recognized in the protein production of prokaryote [20,21], yeast [22], and Chinese hamster ovary (CHO) cells [17,23], their uses in production in transgenic animal mammary gland bioreactors have not yet been explored; therefore, this is addressed in the present study.

The mutant of tissue-type plasminogen activator (t-PA), which contains only the Kringle-2 domain and the protease domain, has a long half-life in patients and is particularly suitable for bolus administration to treat acute myocardial infarction [24,25]. The molecular structure of recombinant thrombolytic agents that work by converting plasminogen to the natural fibrinolytic agent plasmin is closely related to the availability of dissolved thrombus medicine [26–28]. Accordingly, the establishment of novel and efficient rhPA requires accurate design of its structure.

We aimed to enhance rhPA expression through a cleavable goat β-lactoglobulin (BLG) signal peptide in the milk of transgenic rabbits, as well as to improve the molecular structure and biological characteristics for better efficacy and lower side effects as a therapeutic drug. The expression level of rhPA has been reported previously [24,29], but we did not realize that the signal peptide contributed to the high expression level and did not mention it at that time. And the molecular structure and biological characteristics remain unknown. To accomplish this, purification and characterization of rhPA were conducted to identify end-terminal amino acid sequences, molecular mass, and allergic reactions to estimate the effects of signal peptides on rabbit mammary gland-specific expression. The overall results presented herein indicate that insertion of the cleavable goat BLG signal peptide sequence into the expression vector should be beneficial for expressing recombinant proteins in the milk of transgenic animals.

## Materials and methods

### Animals and ethics statement

**Figure 1:**
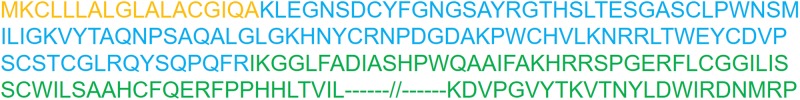
Theoretical amino acid sequence of the cDNA The amino acid sequence of the goat β-casein signal peptide is shown in orange, the amino acid sequence of the K2 domain of recombinant human plasminogen activator is in blue, the amino acid sequence of the P domain of recombinant human plasminogen activator is in green.

The rhPA transgenic New Zealand White rabbit lines were generated as previously reported [24,29]. rhPA transgenic rabbits harbor a mammary gland-specific expression vector that contains an artificial cDNA. The cDNA contained a Kozak translation initiation sequence, a goat BLG signal peptide coding region and the coding sequences of the K2 and P domains of human t-PA (Figure 1). The offspring of founder rabbit K29♂ expressed rhPA in the milk at an average concentration of 1.28 mg/ml.

Guinea pigs weighing 350–400 g were purchased from the Fulai Farm (Nanjing, China; animal license number SCXK(Su)2014-0004).

The rabbits and guinea pigs were kept under controlled conditions (22°C, 55% humidity, and 12 h day-night rhythm) and fed a standard laboratory chow at the College of Veterinary Medicine, Yangzhou University. Animal experiments and procedures were performed in accordance with the guide for the Care and Use of Laboratory Animals (Ministry of Science and Technology of the People’s Republic of China) and approved by the animal care and use committee of Yangzhou University, Yangzhou, China (license number: SYXK(Su)2017-0044).

### Prediction of the presence and location of the signal peptide cleavage site

The theoretical amino acid sequence translated by cDNA (a goat BLG signal peptide coding region and the coding sequence of the K2 and P domains of human t-PA) was analyzed using the SignalP 4.1 Server [30,31] (http://www.cbs.dtu.dk/services/SignalP/) to predict the presence and location of the signal peptide cleavage site.

### Purification of rhPA

The purification process consisted of four steps: pretreatment of transgenic rabbit milk, benzamidine affinity chromatography, cation exchange chromatography, and cibacron blue affinity chromatography. The chromatography experiments were conducted using a protein purification system (ÄKTAprime™ PLUS, GE Healthcare, Sweden) controlled by PrimeView 5.0 (Amersham Biosciences, Sweden).

#### Pretreatment of transgenic rabbit milk

Milk was stored frozen at −80°C before use. For purification, milk was thawed and pretreated by centrifugation at 20000*** g*** for 30 min to get milk whey. The milk whey was then ultrafiltered against Buffer A (25 mmol/L phosphate buffer, pH 7.5) using a 100 kDa ultrafilter (Sartorius, Goettingen, Germany) to remove chyle particles, fat, and macromolecular proteins. The filtrate was then collected for the subsequent benzamidine affinity chromatography.

#### Benzamidine affinity chromatography

The filtrate from the ultrafilter was mixed with Buffer B (3 mol/L NaCl, 25 mM/L Phosphate buffer, pH 7.5) at 5:1, then loaded directly onto a Benzamidine Bestarose 4FF (20.0 × 2.6 cm, BestChrom, China) column equilibrated in Buffer C (0.5 mol/L NaCl, 25 mmol/L phosphate buffer, pH 7.5) in advance. Subsequently, the column was washed with Buffer C and eluted with Buffer D (100 mmol/L glycine, pH 3.0). The eluate was collected for subsequent cation exchange chromatography.

#### Cation exchange chromatography

The eluate of benzamidine affinity chromatography was adjusted to pH 6.5 immediately, and then loaded onto a Capto S ImpAct (5.0 × 1.2 cm, GE Healthcare, Sweden) column equilibrated in Buffer E (25 mmol/L phosphate buffer, pH 6.5) in advance. The column was subsequently eluted with a step gradient of 25% and 27% Buffer F (0.5 mol/L NaCl, 25 mmol/L phosphate buffer, pH 6.5). The eluate with a gradient of 27% Buffer F was then collected for subsequent Cibacron Blue affinity chromatography.

#### Cibacron blue affinity chromatography

The eluate of cation exchange chromatography was diluted five times with Buffer A, adjusted pH to 7.5 and loaded directly onto a Cibacron Blue Sepharose 6 Fast Flow (5.0 × 1.2 cm, GE Healthcare, Sweden) column that had been equilibrated in Buffer A. The column was eluted with a step gradient of 20% and 100% Buffer G (1 mol/L NaCl, 25 mmol/L phosphate buffer, pH 7.5). The eluate with a gradient of 100% Buffer G was collected and desalted using a Bestdex G-25 column (1.6 × 2.5 cm, BestChrom, China) for the subsequent experiments.

### SDS-PAGE and Western blot analysis of rhPA

Samples were boiled at 100°C for 5 min with SDS-PAGE loading buffer, then separated on 12% polyacrylamide Tris-glycine gels. Afterwards, the gels were stained with Coomassie Brilliant Blue G-250, and the purity and concentrations of the samples were determined using the Tanon Gis software (Bio-Tanon, China).

For Western blot analysis, protein samples were resolved by electrophoresis and electro-transferred onto PVDF membrane (0.45 μm, Pall, U.S.A.). The membranes were then blocked with 5% fetal bovine serum/Tris-buffered saline-Tween (TBST) overnight at 4°C, after which they were incubated with mouse monoclonal antibody to the P domain of human t-PA (SC-59721, Santa Cruz Biotechnology, U.S.A.) at 37°C for 1.5 h. The membranes were subsequently incubated with HRP-conjugated goat anti-mouse IgG (Sangon Biotech, China) at 37°C for 1 h, followed by three washes in TBST. Immunodetection was conducted using ECL substrate solution (Millipore Corporation, Billerica, MA, U.S.A.) according to the manufacturer’s instructions.

### Determination of the fibrinolytic activity of rhPA

The fibrinolytic activity of rhPA was assayed using a fibrin agarose plate assay (FAPA) as described elsewhere [24]. Briefly, 1% agarose was brought to a boil, allowed to cool to 45°C, then immediately mixed with fibrinogen and thrombin (37°C). The warm mixture was then dispensed into Plexiglass dishes. When the solution cooled to room temperature, fibrin-thrombin-agarose solidified to a gel state. Sample wells were subsequently drilled in each gel and filled with 20 μl sample solution. In the thrombolytic step, each dish was incubated at 37°C for 12 h. The activity was determined by the diameter size of the thrombin-dissolving ring. A commercially available Alteplase (Actilyse, Boehringer Ingelheim International GmbH, Germany) preparation of recombinant human plasminogen activator (rhPA) was used for comparison with rhPA.

### Structure and molecular weight analysis of purified rhPA

Analysis of the N-terminal amino acid sequence was performed by the Edman degradation reaction using an automated Edman sequencer (PPSQ-31A, Shimadzu, Japan). Briefly, the purified protein was subjected to SDS-PAGE electrophoresis, transferred to a 0.45 μm PVDF membrane (Millipore, Germany) and stained with Ponceau S. The main area of the sample was cut from the membrane and used for sequencing via chemical Edman degradation of the N-terminus. The original data and atlas generated by PPSQ-33A were identified using the PPSQ-30 Data Processing software (Shimadzu, Japan). The identified N-terminal amino acid sequence of purified rhPA was compared with the theoretical sequence data retrieved from the GenBank database (http://www.ncbi.nlm.nih.gov/genbank/) of the National Center for Biotechnology Information.

C-terminal amino acid sequence analysis was performed by liquid chromatography tandem mass spectrometry (LC–MS/MS). Briefly, the sample was digested with chymotrypsin, desalinated and isolated by Ultra Performance Liquid Chromatography (Acquity UPLC I-Class, Waters, U.S.A.), after which it was analyzed by mass spectrometry (Xevo G2-XS QTof, Waters, U.S.A.). The original data analysis was performed using UNIFI (1.8.2, Waters, U.S.A.). The identified C-terminal amino acid sequence of the purified rhPA was compared with that of wild-type human t-PA sequence data retrieved from the GenBank database.

JASCO J-810 spectrometer equipment (Japan Spectroscopic Company, Tokyo, Japan) was used to measure CD spectra of rhPA and Alteplase samples. Proteins (0.1 mg/ml) in 50 mM phosphate buffer (pH 7.4) was measured in the far-UV region. All the data were acquired with an interval of 1 nm and a scan speed of 100 nm/min at 37°C.

The purified rhPA was assayed by matrix-assisted laser desorption/ionization time of flight mass spectrometry (5800 MALDI-TOF/TOF, AB Sciex, U.S.A.), after which the identified molecular weight of the purified rhPA was compared with the theoretically calculated molecular weight.

### ASA analysis of rhPA in guinea pigs

Since rhPA was designed as a pharmaceutical protein, whether the signal peptide changes its immunotoxicity should be analyzed.

A total of 28 guinea pigs weighing 350–400 g were randomly divided into four groups. Animals in each group were then intravenously injected with 0.5 ml negative control (solvent: 0.225 mol/L l-arginine, 0.1114 mol/L phosphoric acid, 0.012% w/v polysorbate 80, pH 7.2), positive control (10 mg/ml bovine serum albumin, A1933, Sigma–Aldrich, Germany + solvent), Alteplase (1.12 mg/ml Alteplase + solvent) or rhPA (1.12 mg/ml rhPA + solvent) at days 1, 3 and 5, and 1 ml of each samples to stimulate at day 14. The symptoms and incidence of systemic allergic responses were assessed after stimulation. Observation and evaluation of anaphylactoid reactions in guinea pigs were conducted based on the guidance on stimulation, anaphylaxis, and hemolysis studies of the CFDA. Blood samples were collected 40 min after stimulation for observation of plasma IgE and histamine contents by the ELISA method (Guinea Pig Immunoglobulin E (IgE) ELISA Kit, Cusabio Biotech Co., Ltd., Wuhan, China, https://www.cusabio.com/; His (Histamine) ELISA Kit, Elabscience Biotechnology Co., Ltd., Wuhan, China). The results of sensitization to guinea pigs were assessed synthetically by observation of allergic response manifestations, as well as serum IgE and histamine contents.

### Statistical analysis

All data are shown as mean ± standard error of the mean (S.E.M.). Statistical significance was determined by one-way ANOVA with the post-hoc Dunnett’s test using the SPSS software (IBM Corporation, U.S.A.). *P* values < 0.05 were considered significant.

## Results

### Prediction of presence and location of signal peptide cleavage site

The theoretical amino acid sequence was analyzed using the SignalP 4.1 Server and predicted based on the euk networks using the SignalP-noTM method. The results indicated that there was a cleavage site between positions 18 and 19: IQA-KL, *D* score = 0.846, *D*-cutoff = 0.450 (Supplementary Figure S1), which matched our expectations.

### Purification of rhPA

**Figure 2:**
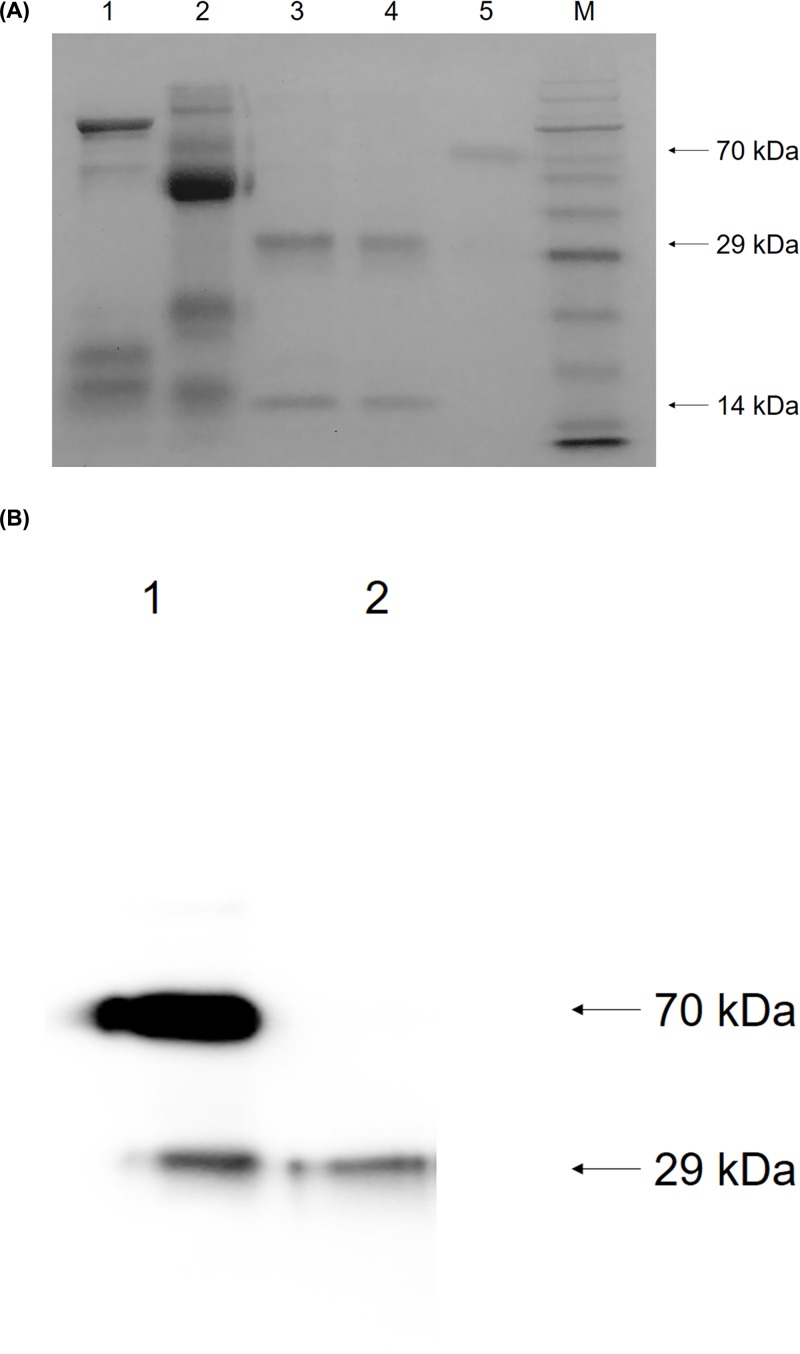
Identification of protein fractions eluted from various chromatography columns (**A**) Identification of purified rhPA by SDS-PAGE. 1: Filtrate from the 100 kDa ultrafilter; 2: eluate of benzamidine affinity chromatography; 3: eluate of cation exchange chromatography; 4: eluate of cibacron blue affinity chromatography; 5: commercially available Alteplase (Actilyse, Boehringer Ingelheim International GmbH, Germany); M: RealBand Pink Blue Protein Marker (Sangon Biotech, China). (**B**) Identification of purified rhPA by Western blot. 1: Commercially available Alteplase, two-chain form; 2: purified rhPA.

The milk from female offspring of K29♂ was pooled and used in the present study. The rhPA was purified in four steps: pretreatment, benzamidine affinity chromatography, cation exchange chromatography, and cibacron blue affinity chromatography. We utilized a high speed centrifuge to obtain milk whey, which was then subjected to ultrafiltration by a 100 kDa ultrafiltrater to remove chyle particles, fat, and macromolecular proteins to prepare the samples for chromatography. Because of the large volume of the filtrate, rhPA was invisible in lane 1 of Figure 2A. A benzamidine affinity chromatography that specifically bound serine proteases was selected for the first chromatography because rhPA is a serine protease. As shown in lane 2 of Figure 2A, the majority of the lactoproteins appeared in the flow-through, while rhPA eluted with Buffer D. Lactoproteins that co-eluted with rhPA were separated from rhPA by subsequent cation exchange chromatography. The results of SDS-PAGE indicated that the eluate of cation exchange chromatography contained an approximately 130 kDa protein (lane 3, Figure 2A). Therefore, cibacron blue affinity chromatograph binding akinases was selected to purify rhPA since rhPA is an akinase. Two bands of approximately 14 and 29 kDa were visualized as shown in lane 4 of the SDS-PAGE (Figure 2A), which indicated that the purified rhPA was in a two-chain form.

The purity was determined to be 98% by scanning the SDS-PAGE gel with a densitometer. The purification process provided an overall rhPA yield of 52% as measured by ELISA.

After SDS-PAGE analysis, the gel was transferred to PVDF membrane by the electro blotting method. The transformation was then immunodetected using antibodies that specifically bound to the P domain of human t-PA, and a single band indicated the presence of rhPA, which was similar to the two-chain form of Alteplase (Figure 2B).

### Fibrinolytic activity of purified rhPA

**Figure 3:**
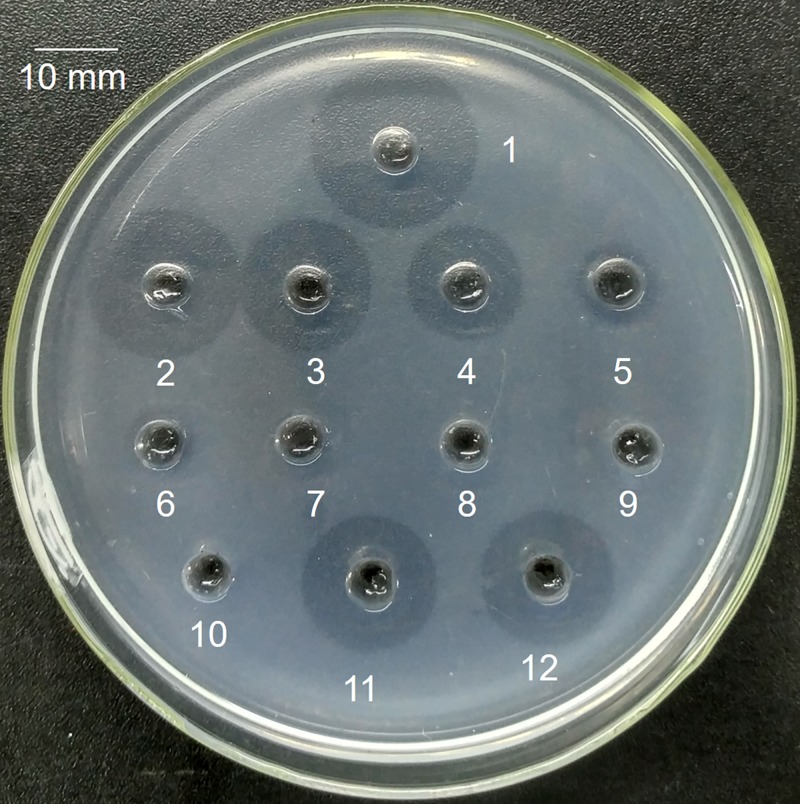
Identification of fibrinolytic activity of the purified rhPA Wells were filled with 20 μl aliquots of samples. Wells 1–7: Alteplase 1000, 500, 250, 125, 62.5, 31.25, and 0 ng/μl with circle diameters of 20, 18, 16, 14, 9, 8, and 6 mm, respectively; 8: null; 9, 10: milk samples of wild-type rabbits diluted 40 times with Buffer A; 11: 3 ng/μl of purified rhPA, circle diameter = 16 mm; 12: milk sample of transgenic rabbits diluted 40 times with Buffer A, circle diameter = 16 mm.

rhPA is a serine protease that converts plasminogen into plasmin, which digests fibrin and dissolves fibrin clots. Therefore, FAPA was applied to evaluate the fibrinolytic bioactivity of purified rhPA *in vitro*, and the index was the size of the fibrinolysis transparent circle (Figure 3). Wells were filled with 20 μl of samples. A standard curve (*y* = 0.9962*x*^3^ − 30.419*x*^2^ + 308.27*x* − 978.61) was constructed using the circle diameter (20, 18, 16, 14, 9, 8, and 6 mm) and the Alteplase concentrations (1000, 500, 250, 125, 62.5, 31.25, and 0 ng/μl). Fibrinolysis transparent circles (diameter = 16 mm) were observed around wells containing 30 ng/μl of purified rhPA, 250 ng/μl Alteplase and milk sample of transgenic rabbit (rhPA 1.2 μg/μl as measured by ELISA) diluted 40 times with Buffer A (Table 2). Which means the biological activities of rhPA in the milk and the purified rhPA determined by FAPA were both approximately 8 times higher than that of commercially available Alteplase. These findings demonstrate that rhPA was correctly posttranslationally modified and the non-immunoaffinity chromatographic methods were able to purify rhPA to a high purity and a high fibrinolytic activity.

### Structure and molecular weight analysis of purified rhPA

**Figure 4:**
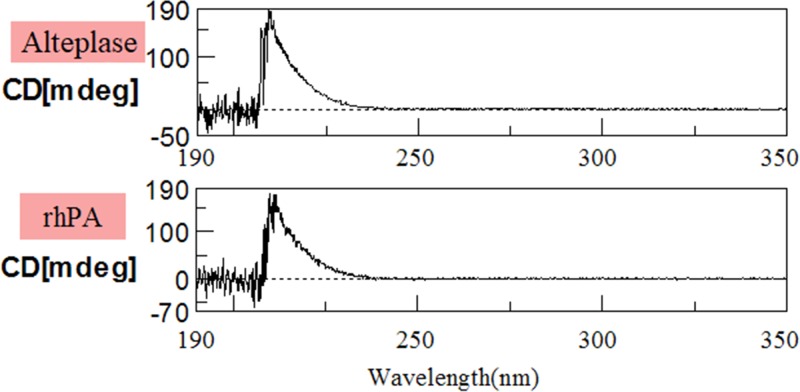
Circular dichroism spectra of Alteplase and rhPA Proteins (0.1 mg/ml) in 50 mM phosphate buffer (pH 7.4) was measured with a 1 mm cell in the far-UV region.

The N-terminal amino acid sequence of the purified rhPA determined by an automated Edman sequencer matched that of the theoretical sequence. The C (cysteine) was incorrectly measured as T (threonine) because of limitations of the test method. The approximately 29 kDa protein was also subjected to sequencing and the sequence matched the P domain of human t-PA (Table 1). The complete sequencing results along with the standards were included in the supplementary material (Supplementary Figures S2 and S3). C-terminal amino acid sequence analyses (Table 1) revealed that the purified rhPA was identical with human t-PA (GenBank: AAO34406.1). As shown in Figure 4, at 210 nm, both rhPA and Alteplase respectively exhibited a positive absorption band. The molecular mass of the purified rhPA was 41713.13 Da as determined by MALDI-TOF/TOF (Table 1).

**Table 1:** Sequencing and molecular mass analysis of purified rhPA

Character	Theoretical/standard	rhPA
N-terminal sequence (K2)	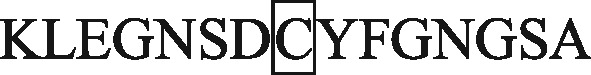	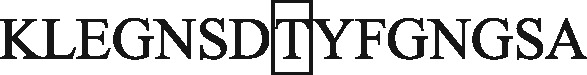
N-terminal sequence (P)	IKGGLFADIASHPWQ	IKGGLFADIASHPWQ
C-terminal sequence	DWIRDNMRP	DWIRDNMRP
Molecular mass	39581.72 Da	41713.13 Da

*Note*. The 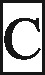 (cysteine) was incorrectly measured as 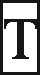 (threonine) because of limitations of the test method.

**Table 2:** FAPA analysis of rhPA

Character	Alteplase (ng/μl)							Null	Milk of WT rabbit diluted 40 times	rhPA (ng/μl)	Milk of transgenic rabbit diluted 40 times (ng/μl)
	1000	500	250	125	62.5	31.25	0	–	–	30	30
Circle diameter (mm)	20	18	16	14	9	8	6	6	6	16	16
Activity (IU/μl)	580	290	145	72.5	36.3	18.1	0	0	0	145	145

The N- and C-terminal protein sequence and molecular mass of rhPA indicated that the purified protein was intact. Additionally, the N-terminal sequence indicated that the goat BLG signal peptide was accurately cleaved from the rhPA preprotein by endogenous rabbit signal peptidase, and that it matched the SignalP 4.1 Server predicted signal peptide cleavage site. CD Spectroscopy revealed that addition of BLG signal peptide to the rhPA has not altered major changes of its secondary structure compared with the native protein. Moreover, when compared with its theoretical molecular mass, there was a slight difference value of 2131.31 Da. The cause of the difference between the theoretical and actual molecular weight may be two possible, suggests one of reason that the rhPA protein expressed in milk is likely to be post-translational modified [32], which determine the functions of proteins. The second possible cause is errors in measurement.

### Active systemic anaphylaxis analysis of rhPA in guinea pigs

**Figure 5:**
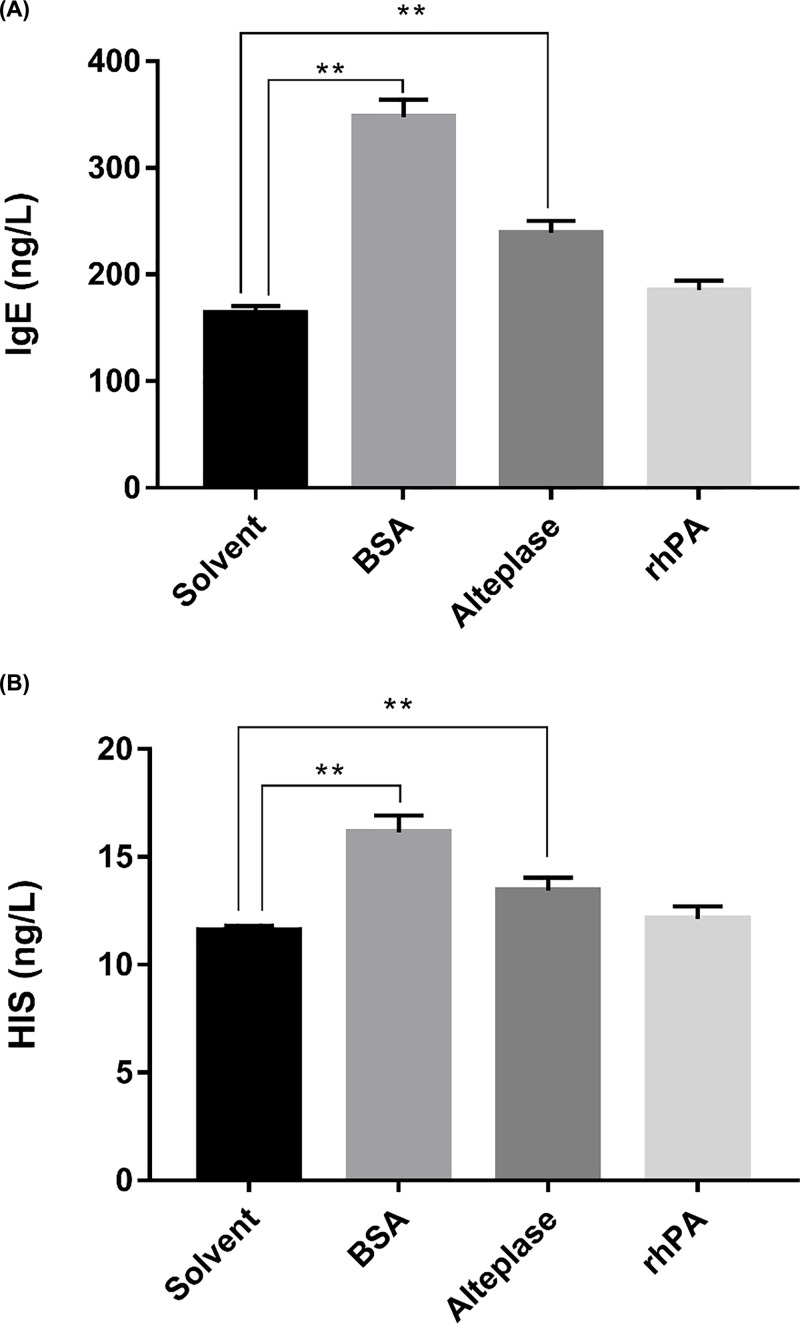
Changes in IgE and histamine in each group of guinea pigs after stimulation (**A**) Changes of IgE in guinea pigs after stimulation. (**B**) Changes of histamine in guinea pigs after stimulation. *N*=7 for solvent (0.225 mol/L l-arginine, 0.1114 mol/L phosphoric acid, 0.012% w/v polysorbate 80, pH 7.2), BSA (10 mg/ml bovine serum albumin, A1933, Sigma–Aldrich, Germany + solvent), Alteplase (1.12 mg/ml Alteplase + solvent), and rhPA (1.12 mg/ml rhPA + solvent). Data are expressed as the mean ± S.E.M. *P*-values were determined by one-way ANOVA followed by a post-hoc Dunnett’s test. **P<0.01 vs negative group.

After stimulation, no significant abnormalities or anaphylactic reactions were observed in the negative control, rhPA, or Alteplase groups. However, positive anaphylactic reactions such as instability of gait, leaping, panting, convulsions, circumgyration, and Cheyne–Stokes breathing were observed in guinea pigs of the positive control group, and five guinea pigs died in 3 min. Serum IgE and histamine were measured using commercial ELISA kits. Although the IgE and histamine levels of the rhPA group were both slightly higher than those of the negative control group, there were no significant differences. The IgE (Figure 5A) and histamine (Figure 5B) levels of the positive control were significantly higher than those of the other groups (*P*<0.01). The active systemic anaphylaxis (ASA) reactions of the purified rhPA were negatively synthetically assessed based on observation of allergic response manifestations and serum IgE and histamine contents.

## Discussion

The results of the present study confirmed those of previous studies that showed the level of proteins secreted can be significantly influenced by replacing the signal peptide sequence with that of a heterologous protein or with a synthetic signal peptide [17–19,22,23]. To improve the efficiency of rhPA gene translation and secretion in the milk of transgenic rabbits, we employed the signal peptide of goat BLG instead of t-PA because the BLG signal peptide can be adapted to guide translation of mRNA in mammary gland epithelial cells. Both the goat BLG signal peptide coding region and coding sequence of the K2 and P domains of t-PA were cloned into a mammary gland-specific expression vector. As expected, transgenic rabbits harboring the vector expressed rhPA in the milk at an average concentration of 1.28 mg/ml, and the concentration of rhPA in the whole lactation period of rabbits were stable (previously reported) [24,29]. Moreover, FAPA revealed that the purified rhPA had high fibrinolytic activity, and did not cause anaphylaxis by ASA which is essential for further therapeutic use.

The utilization of highly heterologous secreted lactoprotein signal peptides to guide the recombinant proteins to translocation and secretion in mammary gland bioreactors has rarely been reported. The first step in the synthesis of secretory proteins is generation of a signal peptide containing 15–30 hydrophobic amino acid residues in the cell matrix. When the polypeptide chain extends to approximately 80 amino acids, the signal peptide directs the synthesizing polypeptide chain into the lumen of the ER. The signal peptidase on the endoplasmic reticulum (ER) then cleaves the signal peptide and continues to extend the polypeptide chain until synthesis of the entire polypeptide chain is completed [33–35]. The translocation of secretory proteins into the lumen of the ER constitutes the limiting step within the classical secretory pathway [36]. The secretion of t-PA from vascular endothelial cells to blood is mediated by its native signal peptide at a low yield [37,38]. However, we report here the secretion of a human recombinant protein in the mammary gland of a transgenic rabbit that was highly efficiently mediated by an accurately cleavable heterologous signal peptide of highly secreted goat BLG. Moreover, the purified rhPA was of high fibrinolytic activity and did not cause ASA reactions. These results indicate that the goat BLG signal peptide can also guide translating ribose to adhere to members of the ER in rabbit mammary gland cells, the signal recognition particle (SRP) in rabbit cells can identify the goat BLG signal peptide, and the goat BLG signal peptide highly efficiently mediated rhPA secretion into milk and was accurately cleaved off from rhPA by the endogenous rabbit signal peptidase, even though rabbit milk does not contain lactoglobulin.

The potential posed by utilizing heterologous signal peptides from highly secreted lactoproteins to improve recombinant protein yields in the milk of transgenic animals is attractive and the identification of a universally applicable strategy involving the use of a single signal peptide for all recombinant proteins would constitute an ideal scenario. It is not unheard of to use signal peptides of lactoproteins to induce secretion of the recombinant protein into milk [39,40]. However, researchers have not analyzed whether the expression level increased or the signal peptide was cleaved. The only drawback to such a case is that it is very difficult to generate transgenic rabbits harboring the same coding sequence in the same integration site with the same copy numbers, but with different signal peptides. To our knowledge, as shown in Table 3, there has rarely been reports of t-PA or its mutants expressed in milk of transgenic animals (especially in rabbits) higher than this report (1130–3660 μg/ml, average 1280 μg/ml). Therefore, we think the signal peptide is a factor in determining the expression level of t-PA (or its mutant) in the milk of transgenic animals.

**Table 3:** Expression of recombinant t-PA (or its mutant) in the milk of transgenic animals

Host animal	Recombinant protein	Signal peptide	Expression (μg/ml)	Ref.
Rabbit	rhPA	BLG	1130–3660	This report
Rabbit	t-PA	Native	0.2–0.85	[41]
Rabbit	t-PA	Native	0.2–0.5	[42]
Rabbit	t-PA	Native	0.05	[43]
Mouse	t-PA	Native	3300	[44]
Mouse	t-PA	Native	3.66	[45]
Mouse	La-tPA	Native	0.18	[46]
Mouse	La-tPA	Native	6	[47]
Goat	La-tPA	Native	3	[48]
Goat	La-tPA	Native	1000–3000	[49]

In the present study, rhPA was produced by a rabbit mammary gland bioreactor, which has advantages in that it has a natural molecular structure, is produced in sufficient amounts and is safe [50]. However, milk is a challenging feedstock for protein purification as it is a multi-phase mixture [51]. Many studies have investigated purification of t-PA and its mutants, but most of these have focused on whether t-PA or its mutants could be expressed by prokaryotes [52–54], CHO cells [55], or plants [56–58], but these purification methods do not work well in the purification of t-PA or its mutants from the milk of rabbits. When purifying t-PA or its mutants in the milk of transgenic animals, immunoaffinity chromatography is useful because of its high specificity [59,60]. Moreover, we and other researchers previously reported the purification of t-PA (or its mutant) using immunoaffinity chromatography alone or combined with gel filtration chromatography or hydrophobic interaction chromatography [59–61]. But immunoaffinity chromatography is expensive and time consuming, the purified proteins are often inactivated during elution [62,63], and the leakage of antibodies or their fragments may cause contaminations in products and then immunogenicity during treatments [64]. Nevertheless, we describe here the use of commercially available benzamidine affinity chromatography, cation exchange chromatography and cibacron blue affinity chromatography for effective purification of rhPA from the milk of a transgenic rabbit.

## Conclusions

The results of the present study reinforce the importance of the rhPA coding region fused to a cleavable heterologous signal peptide from highly secreted goat BLG for improving recombinant rhPA expression. Moreover, the purification and characterization of rhPA are described. It is anticipated that the results presented herein will be widely applied to improve the production of recombinant proteins of significant importance to many areas of biological research, as well as the biopharmaceutical industry.

## Abbreviations

ASA: active systemic anaphylaxis
BLG: β-lactoglobulin
CHO: Chinese hamster ovary
ER: endoplasmic reticulum
FAPA: fibrin agarose plate assay
rhPA: recombinant human plasminogen activator
TBST: Tris-buffered saline-Tween
t-PA: tissue-type plasminogen activator
